# Genome-Wide Detection of Genetic Diversity and Genomic Structure in Hanwoo Cows

**DOI:** 10.3390/ani16203320

**Published:** 2026-10-21

**Authors:** Seungwon Oh, Duhak Yoon

**Affiliations:** 1 Department of Animal Science and Biotechnology, Graduate School, Kyungpook National University, Sangju 37224, Republic of Korea; swoh5250@knu.ac.kr; 2 Research Institute for Innovative Animal Science, Kyungpook National University, Sangju 37224, Republic of Korea

**Keywords:** genetic diversity, Hanwoo cow, linkage disequilibrium, runs of homozygosity, single nucleotide polymorphism

## Abstract

This study aimed to utilize genomic information to elucidate the genetic diversity and structure of Hanwoo cows and develop a more efficient improvement system. Genomic DNA was extracted from hair root samples of 7273 Hanwoo cows, and genotypes were determined using a customized Illumina Hanwoo SNP50k BeadChip. A total of 41,884 single-nucleotide polymorphisms (SNPs) covering approximately 2500 Mb of the genome were selected for analysis, along with 7166 Hanwoo cows that passed quality control. The mean observed and expected heterozygosity were 0.358 ± 0.125 and 0.357 ± 0.124, respectively. A total of 94,560 runs of homozygosity (ROHs) were observed, with an average of 13.2 per animal. Linkage disequilibrium (LD) was estimated using two parameters, *D’* and *r*^2^, which were found to be 0.381 ± 0.309 and 0.054 ± 0.126, respectively, on autosomes. LD declined sharply from< 50 kb to 50–100 kb. In addition, 1818 haplotype blocks were identified on autosomes, totaling 88.27 Mb (3.53% of the autosomal length). The most common haplotype block size was two SNPs, with 1101 blocks. Finally, the effective population size (Ne) was 2853 at 1691 generations but decreased to 277 at 13 generations. These results provide genomic information for further studies and will be helpful for Hanwoo breeding programs.

## Simple Summary

Hanwoo breeding in South Korea has focused on bulls, leading to frozen semen preference bias and concern about reduced genetic diversity. This study aims to assess the genomic structure and genetic diversity of Hanwoo cows using a customized SNP chip. Estimated effective population size declined over past generations, and ROH-based inbreeding was higher in recent birth cohorts, whereas heterozygosity did not decline. These results provide a scientific foundation for monitoring genetic diversity, breed conservation, and more effective cow breeding systems.

## 1. Introduction

Hanwoo cattle, the native cattle of Korea, include four varieties: brown Hanwoo, brindle Hanwoo, black Hanwoo, and Jeju black. Currently, brown Hanwoo is the most widely raised variety, accounting for approximately 3.1 million heads [1]. Since the 1960s, Hanwoo has played an essential role as a valuable resource for meat production alongside Korean economic growth. Hanwoo breeding programs aim to enhance quality traits, such as marbling and flavor, as well as quantity traits, such as meat yield, to improve Hanwoo meat quality [2]. These breeding programs use performance and progeny testing to select proven bulls, which are assigned Korean Proven Bull Numbers (KPNs) [3]. Semen from these proven bulls is distributed to breeding farms for artificial insemination (AI) of cows. Recently, KPNs have become widely used on commercial farms, raising concerns about preserving Hanwoo genetic diversity. Therefore, greater attention to the genetic contribution of Hanwoo cows may be important for maintaining genetic diversity in the breed.

With the development of high-density genomic maps and the availability of large-scale SNP datasets in cattle [4], numerous studies have investigated population structure and genetic diversity using genomic information, including linkage disequilibrium (LD) and SNP marker data [5,6]. Genetic markers, especially single-nucleotide polymorphisms (SNPs), have been widely employed in cattle genomic studies for diverse applications, including marker-assisted selection [7], quantitative trait loci (QTL) mapping [8], genome-wide association studies (GWAS) [9], genomic selection (GS) [10], and assessment of the accuracy of genomic estimated breeding values (GEBV) [11]. The application of SNP markers has also been extended to other livestock species, including horses [12], pigs [13], and sheep [14]. In particular, a Hanwoo-specific customized SNP chip, the Illumina Hanwoo SNP50k BeadChip (Illumina Inc., San Diego, CA, USA), has been developed for Hanwoo, and this platform has been utilized in genomic prediction and GWASs of economically important traits [15,16]. Despite these advances, genomic characterization of genetic diversity and population structure in Hanwoo cows remains warranted.

Runs of homozygosity (ROHs) are long continuous stretches of homozygous genomic segments that can arise from the inheritance of identical-by-descent (IBD) segments from common ancestors [17]. Long ROHs suggest recent inbreeding within the population, whereas shorter segments indicate more distant relationships with common ancestors [18]. ROH analysis is a useful approach for investigating genomic patterns of homozygosity and population history in cattle and other livestock species [19]. Several studies have used genomic data to estimate ROHs and elucidate the population structure of Hanwoo [3,20].

LD represents the non-random associations of alleles at two loci within a population and can be influenced by factors such as physical distance, allele frequency, genetic drift, and selection [21]. The extent of LD can provide information on historical recombination patterns and Ne, which can contribute to the evaluation of population structure and genetic diversity. Moreover, LD plays a crucial role in determining the accuracy of genomic selection [22].

Haplotype blocks are regions within a chromosome that exhibit low recombination rates and high levels of LD [23,24]. Haplotype blocks are influenced by factors such as recombination, mutation, selection, and population bottlenecks [25]. Haplotype block structure can affect genomic prediction accuracy [26] and contribute to the interpretation of GWAS results [27]. Therefore, understanding the haplotype block structure can provide useful information for interpreting genomic association studies in Hanwoo [23].

Characterizing the genetic diversity and genomic structure of Hanwoo cows is important for developing sustainable breeding strategies and maintaining genetic diversity in the breed. However, genomic studies focusing on Hanwoo cows remain limited. Therefore, this study utilized genomic data from Hanwoo cows to estimate ROHs and analyze LD, haplotype block structure, and Ne. These analyses provide insights into the genetic diversity and population structure of Hanwoo cows and may contribute to the development of appropriate breeding and conservation strategies.

## 2. Materials and Methods

### 2.1. Animal Sampling and Quality Control

A total of 7273 Hanwoo cows were sampled, with cows from 7 regions in Gyeongbuk province selected. All cows were the progeny of 401 sires (18.1 progeny per sire) and 5526 dams (1.3 progeny per dam). Outsourcing facilities obtained consent from each Hanwoo cow farm, collected hair roots, extracted genomic DNA, and genotyped it using the Illumina Hanwoo SNP50k BeadChip (Illumina Inc., San Diego, CA, USA), which contained 53,866 SNPs. The results were provided in PLINK format (ped and map files). Quality control was performed using PLINK v1.9 (Harvard University, Boston, MA, USA) [28]. Only autosomal markers were retained. Samples with a call rate < 0.90 (‘--mind 0.1′) and duplicate samples, defined as pairs with a genomic relationship ≥ 0.9 and a genotype concordance ≥ 0.99 (one cow per pair), were removed. Markers with a call rate < 0.90 (‘--geno 0.1′) and one marker of each pair at an identical position were removed. Parental IDs were then set to zero (‘--make-founders’) so that all 7166 cows were used for all calculations, and markers with minor allele frequency (MAF) < 0.05 (‘--maf 0.05′) were removed.

### 2.2. Genetic Diversity

Genetic diversity was analyzed by calculating observed heterozygosity (H_O_), expected heterozygosity (H_E_), the inbreeding coefficient (F), and MAF. H_O_, H_E_, and F values were obtained for each autosome using the ‘--hardy’ option and F was calculated for each SNP as 1 − H_O_/H_E_. MAF was estimated using the ‘--freq’ option in PLINK v1.9. To show the effect of the MAF filter, MAF was grouped into six categories before filtering: (1) 0–0.05, (2) 0.05–0.1, (3) 0.1–0.2, (4) 0.2–0.3, (5) 0.3–0.4, and (6) 0.4–0.5. H_O_, H_E_, and the mean MAF refer to the final dataset (MAF ≥ 0.05). Genomic relationships among cows were estimated as the variance-standardized genomic relationship matrix (G) with PLINK v1.9 (‘--make-rel square’) from the 41,884 SNPs; the mean of the off-diagonal elements and the proportion of cow pairs with G ≥ 0.25, which corresponds approximately to a half-sib or closer relationship, were determined. To examine temporal trends, H_O_ and sample size corrected H_E_ (*H_E_* × 2*n*/(2*n* − 1), where n is the number of cows in a cohort) were calculated for the 7088 cows with a known birth date, grouped into 11 birth cohorts (≤2011 to 2021; 198–1238 cows per cohort).

### 2.3. ROHs

ROHs were estimated individually for each Hanwoo cow using PLINK v1.9 with the ‘--homozyg’ option. Because the mean marker interval in this panel was 59.7 kb, the default minimum of 100 SNPs would have restricted detection to segments longer than approximately 6 Mb. Therefore, the following thresholds adapted to the marker density were applied [29]: (1) minimum number of consecutive SNPs in ROHs of 30; (2) minimum ROH length of 1 Mb; (3) density of 1 SNP per 200 kb; (4) maximum gap between consecutive homozygous SNPs of 1000 kb; (5) sliding window of 30 SNPs across the genome; (6) maximum of 1 heterozygous locus per window; (7) proportion of homozygous overlapping windows of 0.05. The detected ROHs were categorized into five groups: 1–4, 4–10, 10–20, 20–30, and >30 Mb. For each individual, the total number of ROHs, the mean number of ROHs per cow, and the average length of ROHs were determined. Genomic inbreeding (F_ROH) was computed as the total ROH length divided by the autosomal genome length (2500.1 Mb). The percentage of each chromosome covered by ROHs was obtained as the total ROH length on that chromosome divided by the product of the number of cows and the chromosome length. F_ROH (ROH ≥ 1 Mb and ≥ 4 Mb) was also derived for each birth cohort.

### 2.4. LD

LD estimates the non-random association between two loci. *D*’ and *r*^2^ are the most commonly used parameters for calculating LD [30]. For each chromosome, *D’* and *r*^2^ were estimated for all SNP pairs within 1000 kb (‘--r2 dprime --ld-window 99999 --ld-window-kb 1000 --ld-window-r2 0′). *D*’ was calculated using the following equation [31]:

(1) $$D^{\prime }=\left\{\begin{array}{cc} \frac{D}{min(freq\left(A\right)\times freq\left(b\right),freq(a)\times freq(B)} & ,if\;D>0\\ \frac{D}{min(freq\left(A\right)\times freq\left(B\right),freq(a)\times freq(b)} & ,if\;D<0 \end{array}\right\}$$

and

(2) D=(freq(AB)×freq(ab)−freq(Ab)×freq(aB))

where *freq(A)*, *freq(a)*, *freq(B)* and *freq(b)* are the allele frequencies of SNPs and *freq(AB)*, *freq(ab)*, *freq(Ab)* and *freq(aB)* are the four haplotypes in Hanwoo cow.

*r*^2^ between syntenic SNP markers was calculated using the following equation [32]:

(3) $$r^{2}=\frac{{\left(D\right)}^{2}}{freq(A)\times freq(a)\times freq(B)\times freq(b)}$$

### 2.5. Haplotype Block Structure

Haplotype blocks were estimated for each chromosome with PLINK v1.9 using the ‘--blocks no-pheno-req’ option, following the method described in [23]. The maximum distance between marker pairs was 500 kb (‘--blocks-max-kb 500′). Results were visualized using Haploview v4.2 (Broad Institute, Cambridge, MA, USA) [33], displaying pairwise *r*^2^ values.

### 2.6. Ne

Ne was estimated from the relationship between LD and the recombination rate (*c*) between SNPs [34] using R v4.5.3 [35]. In this study, we calculated Ne using the following equation:

(4) $$N_{T}(t)={\left(4f\left(c_{t}\right)\right)}^{-1}\times \left(E{\left[{r^{2}}_{adj}|c_{t}\right]}^{-1}-\alpha \right)$$

where *N_T_* is the Ne *t* generations ago, *t* is calculated as $t={\left(2\int \left(c_{t}\right)\right)}^{-1}$ [36], *c_t_* is the recombination rate, ${r^{2}}_{adj}$ is the LD value (*r*^2^) adjusted for the sample size and is calculated as ${r^{2}}_{adj}=r^{2}-{\left(\beta n\right)}^{-1}$ (*n* is the sample size and *β* = 1 because genotypes were unphased), and *α* is a correction for the occurrence of mutations [37] and was set to 1. The recombination rate (*c*) was derived from the physical distance between SNPs, which was converted into the linkage distance *d* (in Morgans) assuming 1 Mb = 1 cM, and *c* was calculated as $c=d\left(1-\frac{1}{2}d\right)$ [38]. Only SNPs with MAF ≥ 0.05 on autosomes were used. For this analysis, *r*^2^ was calculated for all SNP pairs within 4000 kb on each autosome using PLINK v1.9 (*‘*--r2 --ld-window 99999 --ld-window-kb 4000 --ld-window-r2 0′), which yielded 2,918,857 SNP pairs. The pairs were grouped into 80 distance bins of 50 kb (each bin containing at least 34,489 pairs), and Ne was calculated for each bin from the mean *r*^2^ and the mean distance of the bin.

## 3. Results

### 3.1. Data Description

In the present study, the numbers of cows and SNPs removed at each quality control step are shown in Table 1. Of the 7273 sampled cows, 7166 (98.5%) and, of the 52,195 autosomal SNPs, 41,884 (80.2%) were retained. These markers spanned 2500.10 Mb of the genome. The fewest SNPs on a chromosome were 730 on chromosome 28, and the most were 2626 on chromosome 1. The longest chromosome was chromosome 1 (158.09 Mb), and the shortest was chromosome 25 (42.65 Mb). The average marker interval was 0.0597 ± 0.0629 Mb.

### 3.2. Estimation of Genetic Diversity

**Figure 1:**
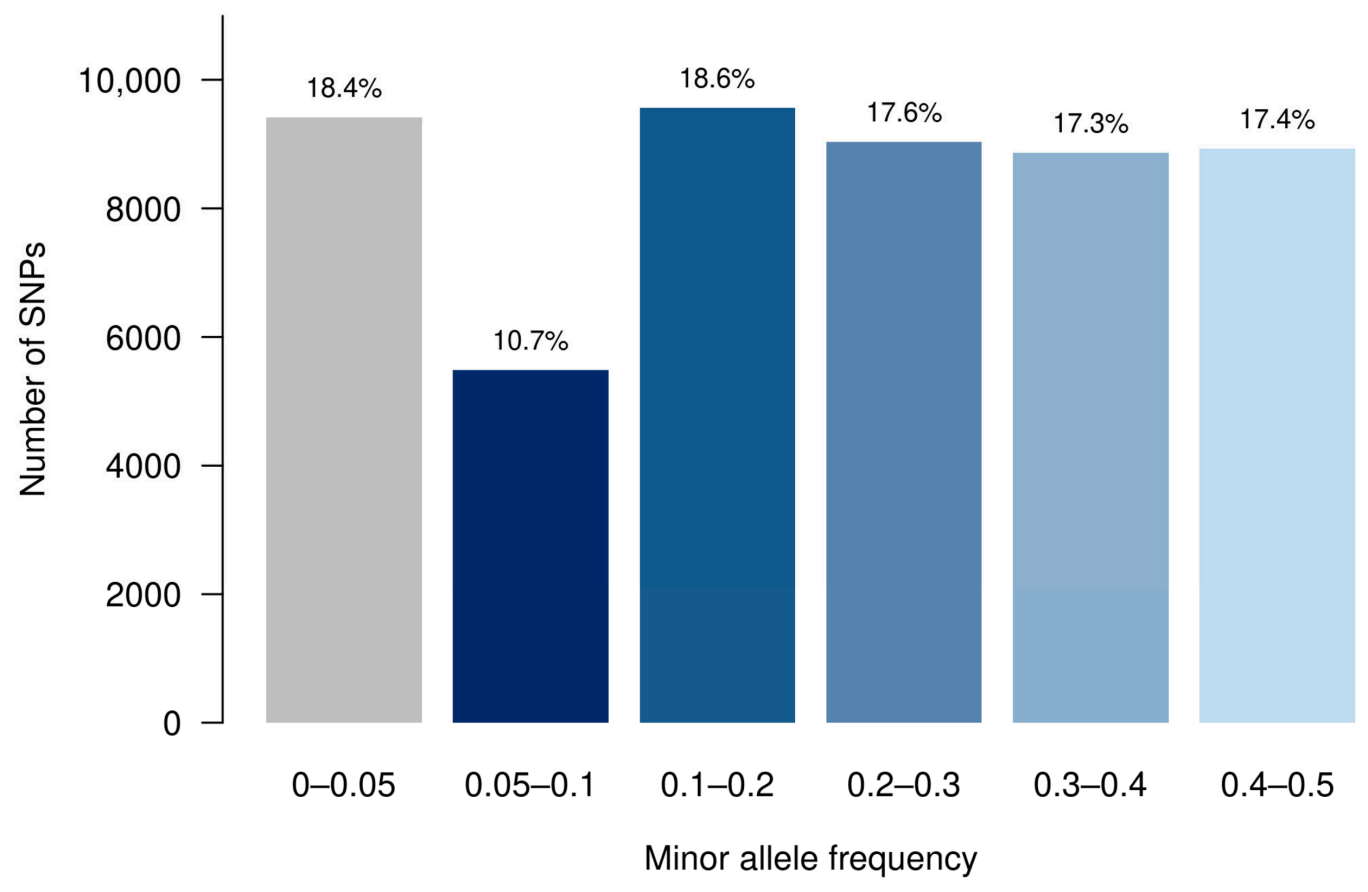
Distribution of MAFs of 51,302 autosomal SNPs in 7166 Hanwoo cows before MAF filtering. Percentages indicate the proportion of SNPs in each MAF class.

The number of SNPs, heterozygosity, and MAF per chromosome are summarized in Table 2. The average number of SNPs was 1444, with H_O_ and H_E_ averaging 0.358 ± 0.125 and 0.357 ± 0.124, respectively. H_O_ and H_E_ were nearly identical, differing by 0.001. The inbreeding coefficient (F) was −0.002 ± 0.034. Before MAF filtering, 18.4% of the SNPs (9418 of 51,302) had an MAF < 0.05 (Figure 1); these SNPs were removed. The mean MAF of the final dataset was 0.268 ± 0.132. The highest average MAF was observed on chromosome 25 at 0.280 ± 0.136, while the lowest was on chromosome 14 at 0.252 ± 0.126. The genomic relationship among the 7166 cows was low on average (mean off-diagonal G = −0.00014), and only 0.29% of the cow pairs had G ≥ 0.25 (approximately half-sib level or closer). Among the 7088 cows with a known birth date, grouped into 11 birth cohorts, H_O_ ranged from 0.352 to 0.359 and sample-size-corrected H_E_ from 0.353 to 0.357. Heterozygosity did not decline over time. H_O_ was 0.352 in the oldest cohort (≤2011) and 0.357 in the 2021 cohort, and H_E_ was 0.353 and 0.356 (Table 3).

### 3.3. Detection of ROHs

**Figure 2:**
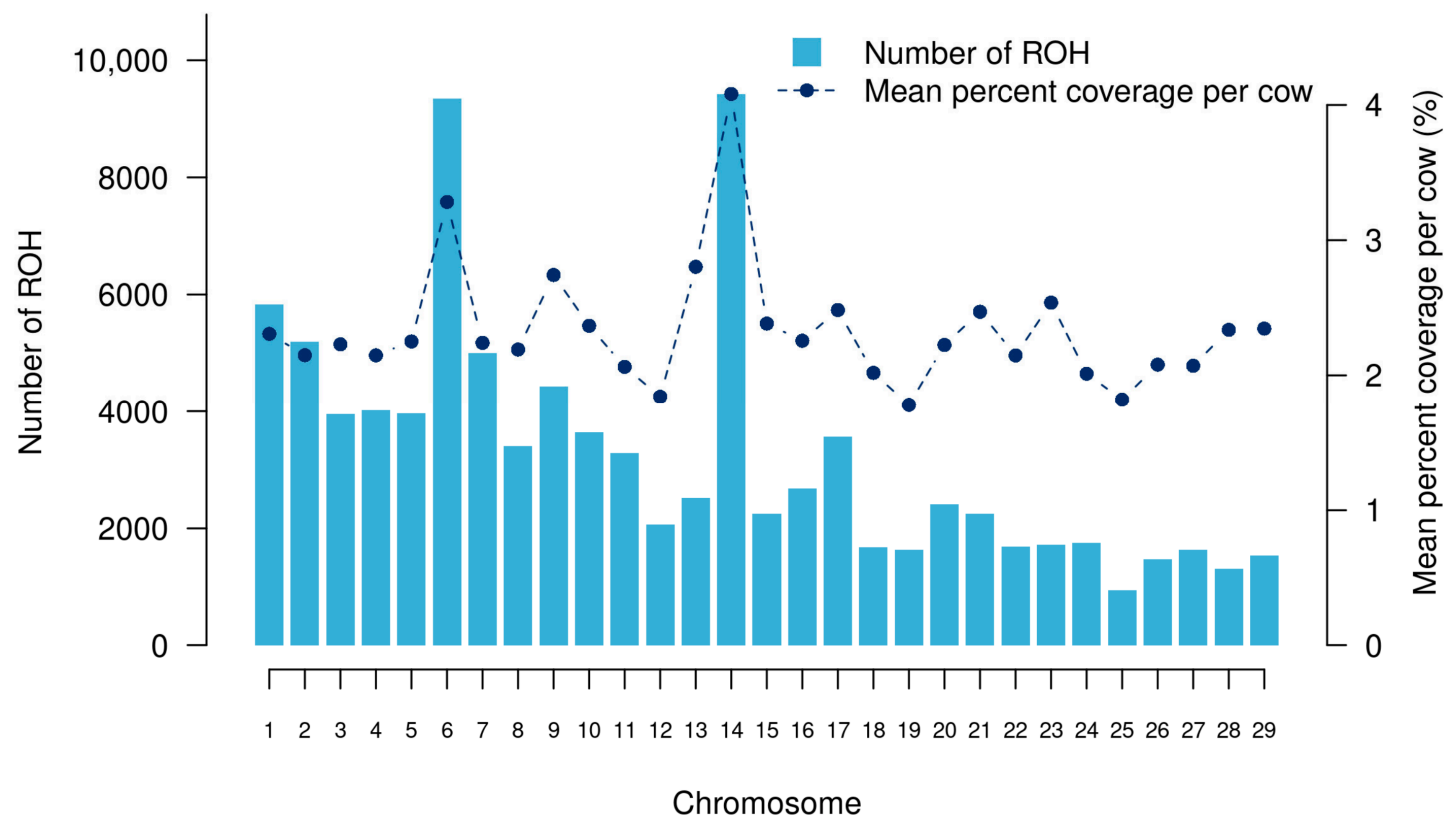
Number of ROHs per chromosome and percent coverage in Hanwoo cows.

**Figure 3:**
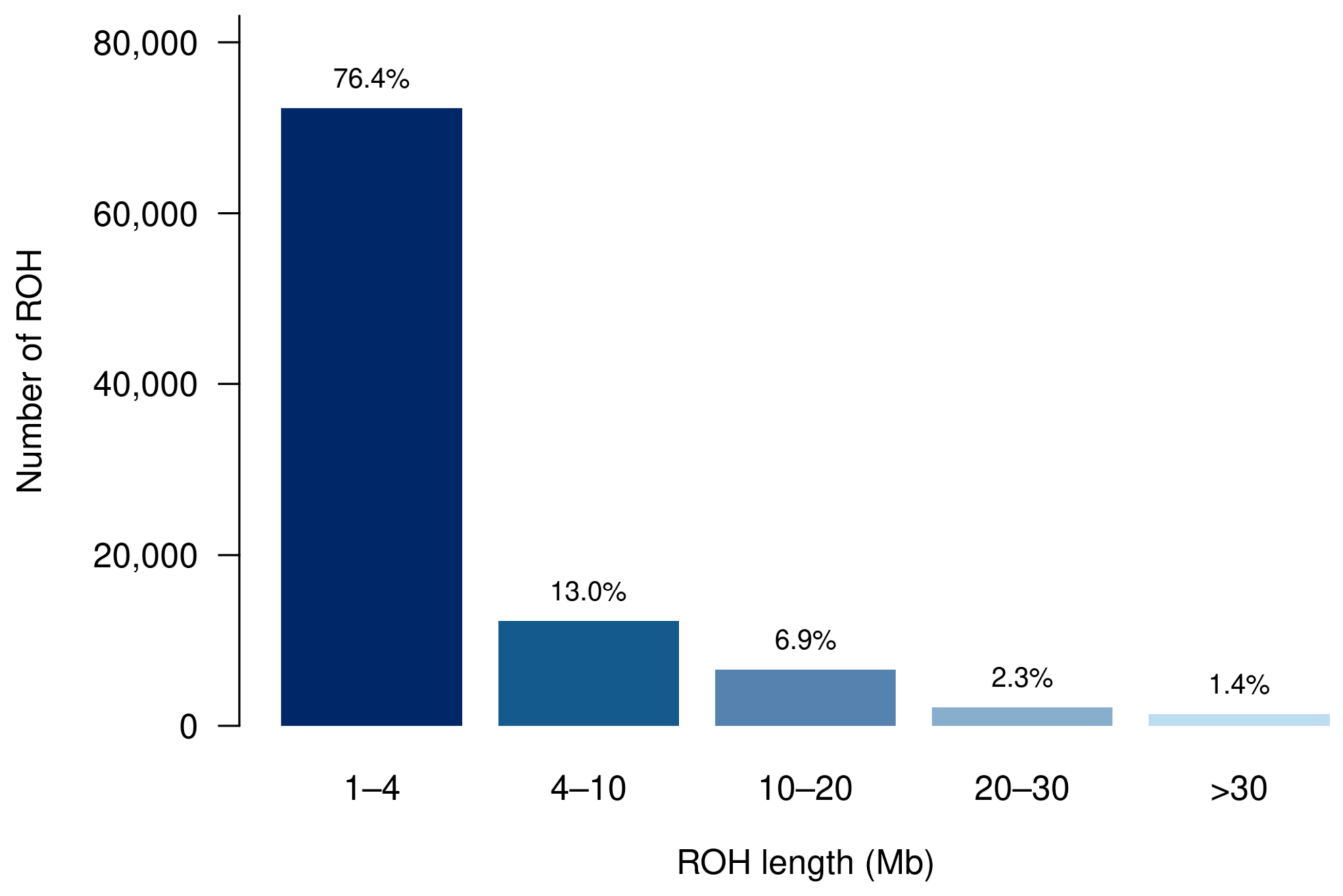
Frequency and proportion of detected ROHs by length.

**Figure 4:**
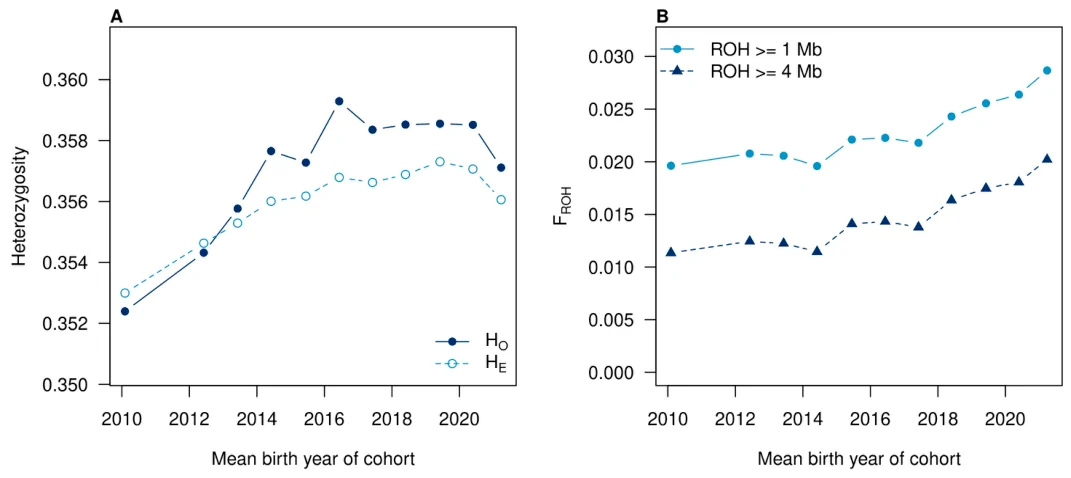
Changes in heterozygosity and F_ROH by birth cohort in Hanwoo cows. (**A**) H_O_ and H_E_; the y-axis is truncated. (**B**) F_ROH calculated from ROH ≥ 1 Mb and ≥4 Mb. Points are plotted at the mean birth year of each cohort.

A total of 94,560 ROHs were detected, averaging 13.2 ROHs per cow; all 7166 cows carried at least one ROH. The mean ROH length was 4.47 ± 6.51 Mb, and the mean F_ROH was 0.024. ROHs were detected on all autosomes (Figure 2). The most ROHs were detected on chromosome 14 (9424), followed by chromosome 6 (9347), and the fewest on chromosome 25 (940). Percent coverage was highest on chromosome 14 (4.08%), followed by chromosome 6 (3.28%). ROH lengths were classified into five categories (1–4, 4–10, 10–20, 20–30, and >30 Mb; Figure 3). Most ROHs measured 1–4 Mb (72,265 ROHs, 76.4%; carried by all 7166 cows), and the number of ROHs larger than 4 Mb decreased. Very few ROHs (1357, 1.4%; 1030 cows) measured over 30 Mb. F_ROH for ROH ≥ 1 Mb increased across birth cohorts from 0.0196 (≤2011) to 0.0287 (2021), and F_ROH for ROH ≥ 4 Mb from 0.0114 to 0.0202 with the largest values in the 2018–2021 cohorts (Table 3, Figure 4).

### 3.4. Estimation of LD

**Figure 5:**
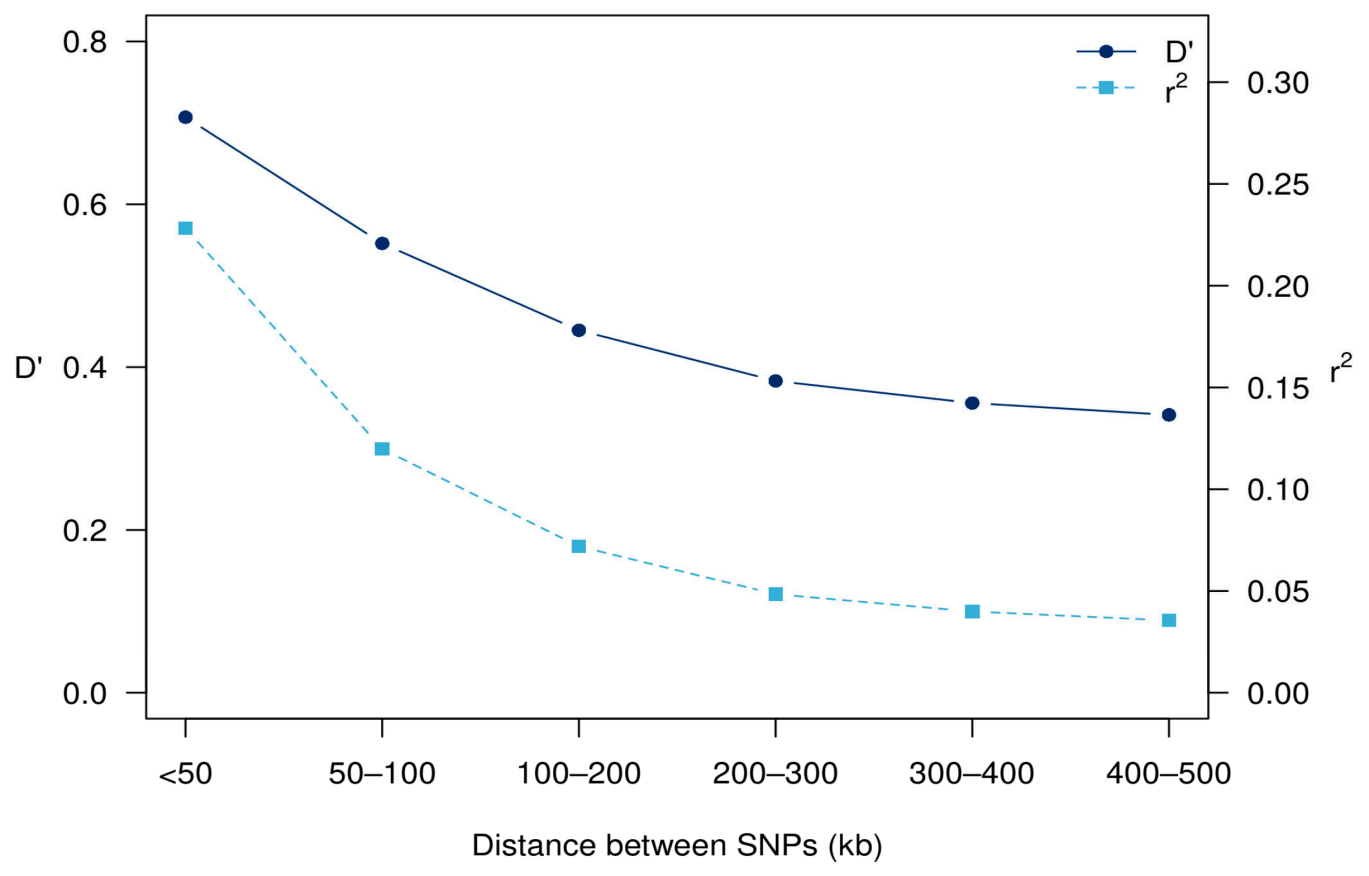
Decay of LD with physical distance between SNPs on autosomes. Mean *D’* (**left axis**) and *r*^2^ (**right axis**) are shown for six distance intervals.

**Figure 6:**
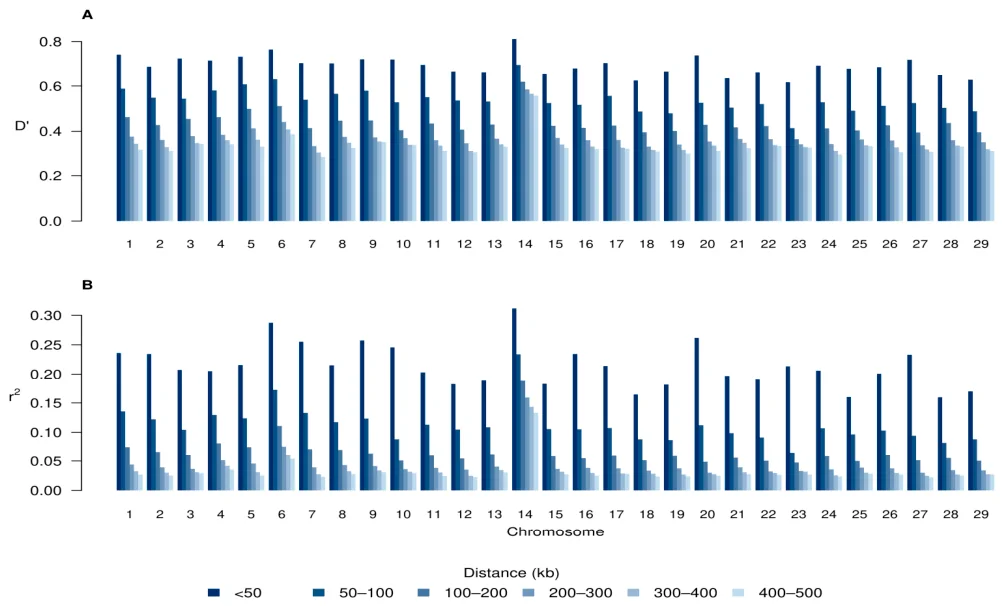
Average LD of each chromosome across distance intervals in Hanwoo cows. (**A**) *D’* and (**B**) *r*^2^. Bars within each chromosome represent the six distance intervals, from the shortest (darkest) to the longest (lightest).

Table 4 summarizes the number of SNP pairs by autosome within 0–1000 kb and the average *D’* and *r*^2^ for each chromosome. The total number of SNP pairs was estimated to be 777,565, with a maximum of 68,475 on chromosome 6. The average *D’* and *r*^2^ values were 0.3807 ± 0.3093 and 0.0538 ± 0.1260, respectively. The highest values of *D’* (0.5759 ± 0.3593) and *r*^2^ (0.1510 ± 0.2414) were observed on chromosome 14. Additionally, the lowest value of *D’* (0.3270 ± 0.2803) was on chromosome 7, and the lowest value of *r*^2^ (0.0364 ± 0.0869) was on chromosome 12 (Table 4). LD decay of the two parameters over six distance intervals (<50, 50–100, 100–200, 200–300, 300–400, and 400–500 kb) is shown in Figure 5. The value of *D’* decreased from 0.707 at a distance of <50 kb to 0.552 at 50–100 kb and finally to 0.341 at 400–500 kb. Similarly, the value of *r^2^* decreased from 0.228 at a distance of <50 kb to 0.120 at 50–100 kb and eventually to 0.036 at 400–500 kb (Figure 5). Figure 6 shows the average *D’* and *r*^2^ of each chromosome in these intervals. Both parameters decreased with increasing distance on every chromosome, and chromosome 14 remained the highest across the intervals. In the <50 kb interval, the average *r^2^* exceeded 0.2 on 19 of the 29 chromosomes, with the highest value of 0.312 on chromosome 14 (Figure 6B).

### 3.5. Characteristics of Haplotype Block Structure

**Figure 7:**
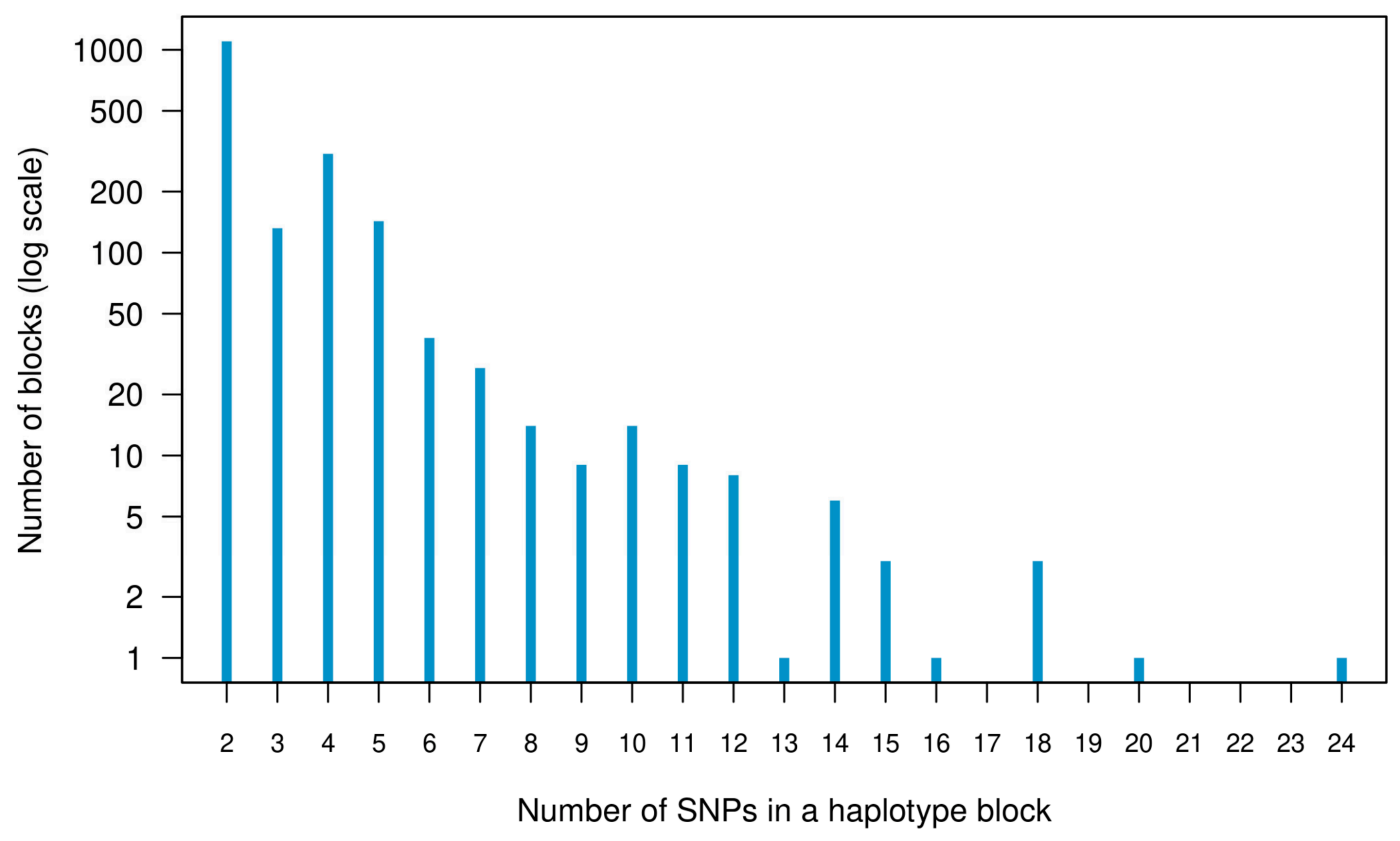
Number of SNPs per haplotype block in Hanwoo cows.

**Figure 8:**
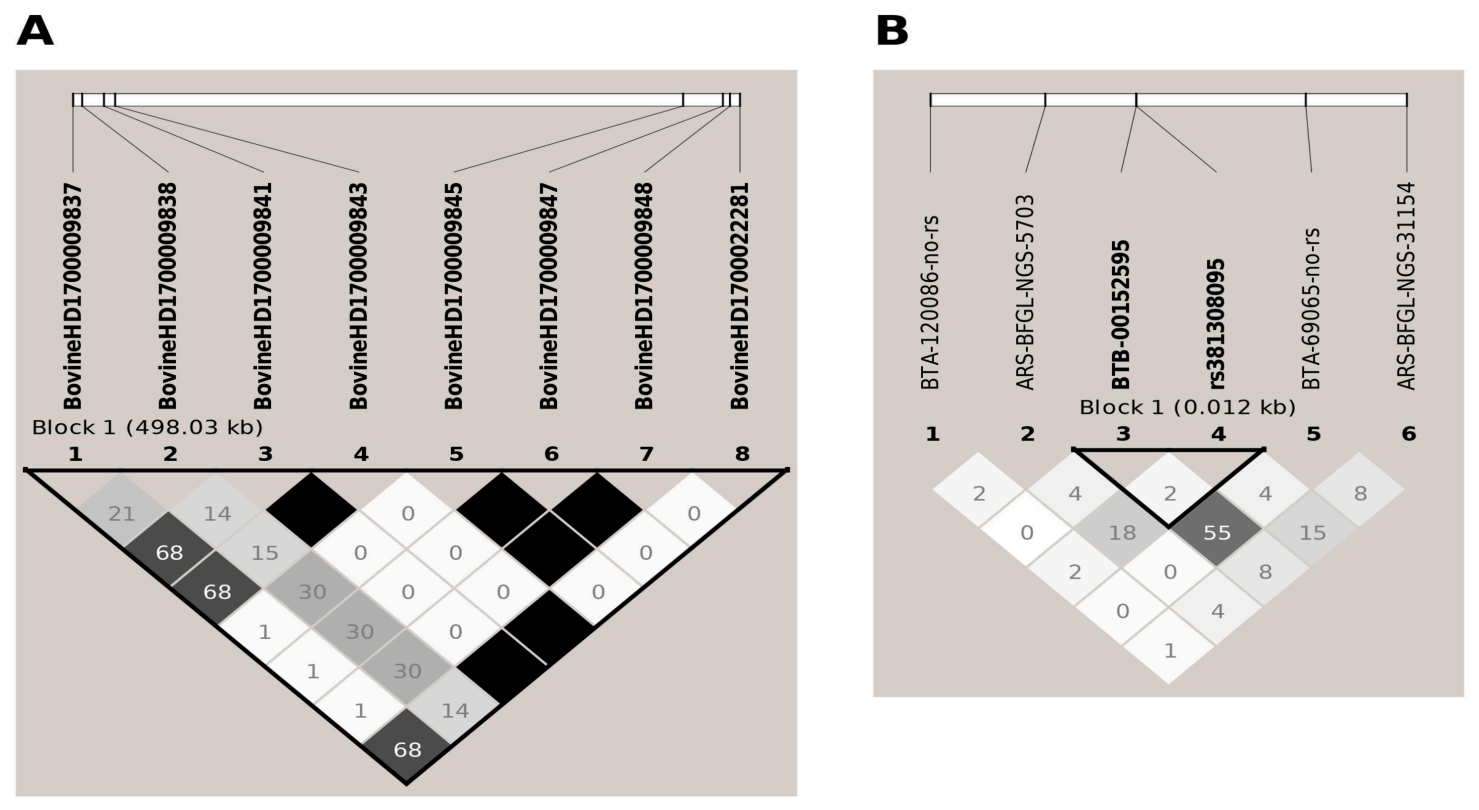
Haploview LD (*r*^2^) plot. (**A**) The longest haplotype block (~498 kb) on chromosome 17. (**B**) The shortest haplotype block (~0.012 kb) on chromosome 3. Shading indicates pairwise *r^2^* between SNPs (white, *r^2^* = 0; grey, 0 < *r^2^* < 1; black, *r^2^* = 1), and numbers in the diamonds are *r^2^* × 100. Haplotype blocks are outlined by black triangles.

The distribution of genome-wide haplotype block structures is presented in Table 5. A total of 1818 haplotype blocks covering 88.27 Mb (3.53% of the autosomal genome) were detected. The average length of the haplotype blocks was 48.55 ± 75.32 kb. The largest number of haplotype blocks was 245, with a total block length of 13,176.41 kb (0.53% of the autosomal genome) on chromosome 6. These blocks had an average size of 53.78 ± 80.61 kb and were composed of 844 SNPs. The largest average block size was 70.997 ± 76.01 kb on chromosome 8. The average number of SNPs forming haplotype blocks was 3.16 ± 2.11, with a maximum of 24 SNPs on chromosome 5 and a minimum of 2. The number of SNPs located in blocks ranged from 32 on chromosome 28 to 844 on chromosome 6. There were 1101 haplotype blocks with 2 SNPs (60.6%) and 307 haplotype blocks with 4 SNPs (Figure 7). The longest haplotype block was 498.03 kb on chromosome 17 and included eight SNP markers, while the shortest was 0.012 kb on chromosome 3 and included two SNP markers (Figure 8).

### 3.6. Past Ne

**Figure 9:**
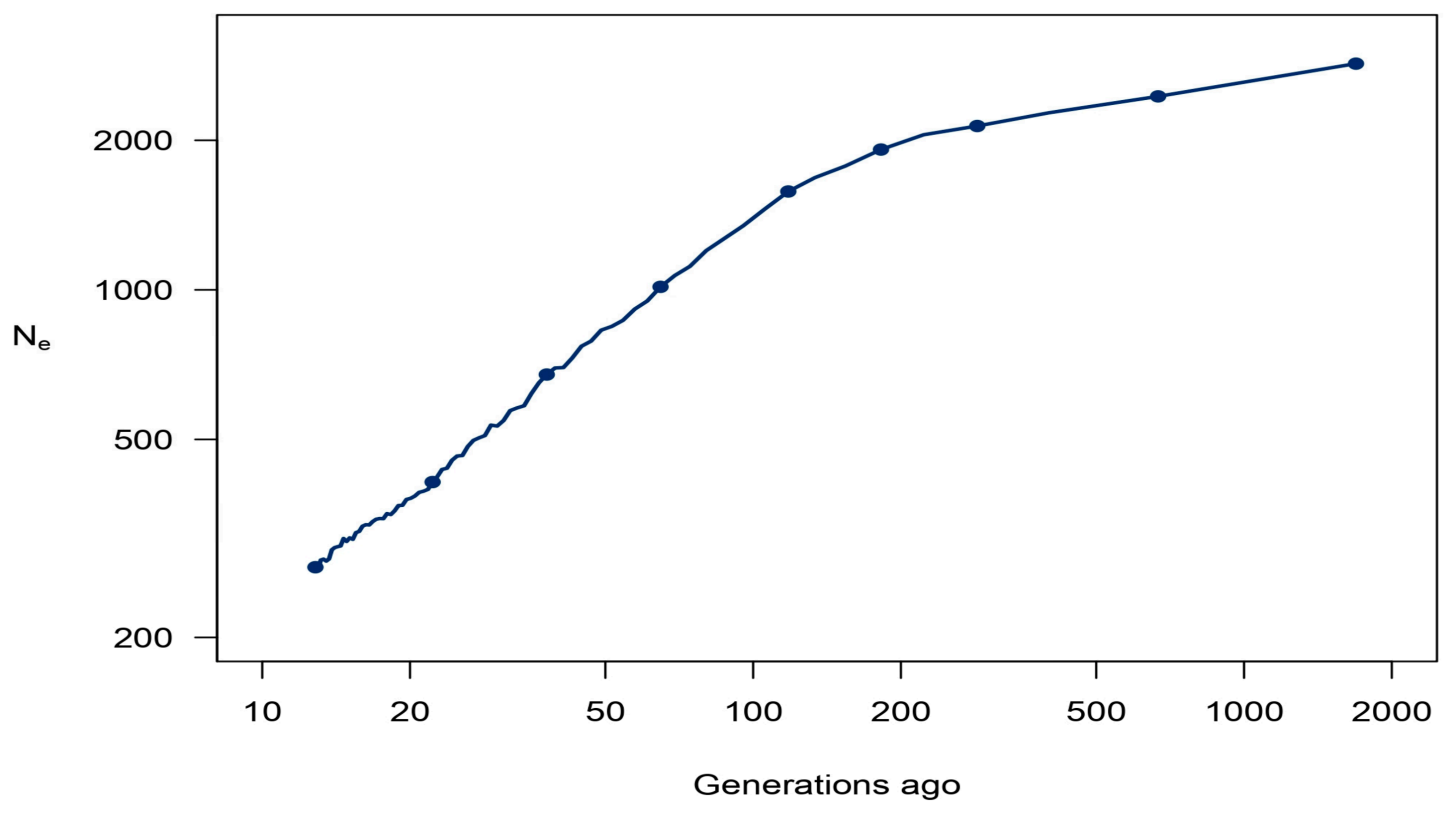
Estimated Ne over past generations using LD (*r*^2^). Both axes are on a log scale.

To assess the magnitude of size change in the cow population from the past to the present, past Ne was estimated using the extent of LD (*r*^2^). Ne decreased from 2853 (1691 generations ago) to 277 (13 generations ago), a decrease of about 10-fold (Figure 9). The decline was gradual until approximately 180 generations ago (Ne = 1916 at 182 generations ago) and became steeper thereafter (1346 at 95, 830 at 49, and 381 at 20 generations ago).

## 4. Discussion

To our knowledge, this study represents the first to estimate the genetic diversity, ROHs, LD, haplotype blocks, and Ne in a large population of Hanwoo cows using a customized Hanwoo SNP50k BeadChip. Some studies have previously examined LD [39], ROHs [3], haplotype block structure [40], and Ne [41] in cattle using Hanwoo genomic data. In particular, the study by [42] was the first to estimate LD and Ne in Hanwoo using low-density SNP arrays. As SNP arrays continue to advance, studies using these arrays are increasingly common [20,43]. In South Korea, the Hanwoo SNP50k BeadChip was developed and is used in Hanwoo breeding programs. Moreover, many studies have been conducted in various cattle breeds using diverse SNP arrays [44,45,46,47].

The genetic parameters (H_O_, H_E_, F, and MAF) were used to evaluate the level of genetic variation within the Hanwoo cow population. An H_O_ value of 0.37 ± 0.12 was reported using the Bovine SNP50k BeadChip [40], close to the value obtained in this study (difference of about 0.01), although the two chips differ. Another cattle breed study estimated the inbreeding coefficient of Hanwoo cattle from SNP markers as −0.04 [48]; our estimate (−0.002) is closer to zero, but a direct comparison is difficult because of the large difference in sample size (18 vs. 7166). The MAF values above 0.05 were distributed fairly evenly, as in [40]. The F value close to zero indicates that observed and expected heterozygosity were almost identical, that is, no overall excess or deficit of heterozygotes.

Genomic relationships among the cows were low on average, but a small fraction of pairs (0.29% with G ≥ 0.25) were closely related. H_O_ did not decline across birth cohorts, whereas F_ROH increased from 0.0196 to 0.0287, which suggests a modest rise in ROH-based inbreeding whose cause cannot be determined from these data. These cohort comparisons are descriptive because the cohorts differed in size, the older cohorts include only cows alive at sampling, and all cows came from one province.

ROHs occur in all breeds but vary in length and frequency by breed. In Hanwoo cattle, short ROH segments were common across three distinct breeds [20], consistent with the findings of the current study. Similar patterns were reported across various cattle breeds examined for ROHs utilizing SNP chips [49,50,51]. Both inbreeding and selection pressures influence the presence of ROHs. Long ROHs suggest recent inbreeding, whereas short ROHs reflect more distant common ancestors [18,52]. In this study, 76.4% of ROHs were 1–4 Mb and 23.6% were ≥4 Mb (22,295 ROHs), which suggests that most ROHs trace back to distant common ancestors rather than to recent common ancestors; nevertheless, ROHs ≥ 4 Mb were found in most cows (5377 of 7166 cows carried ROHs of 4–10 Mb) and F_ROH for ROH ≥ 4 Mb increased in recent birth cohorts (Table 3). Chromosomes 6 and 14 had the most ROHs (9347 and 9424) and the highest coverage (3.28% and 4.08%). QTLs for growth and carcass traits have been reported on these chromosomes in Hanwoo [16,53], but selection was not tested in this study, so the overlap should be regarded as a hypothesis for further study. Currently, most agricultural operations in South Korea use a limited set of KPNs for AI applications [3]. Further validation involving larger populations and a broader range of KPNs is necessary to determine the potential occurrence of inbreeding.

One factor affecting the ROH detection is the density of SNP chips used in ROH studies [54]. A high frequency of short ROHs may be overestimated when a medium-density chip (50K) is used [55]. In this study, the mean marker interval was 59.7 kb and 76.4% of ROHs were 1–4 Mb, so part of the short ROHs may represent homozygous stretches that are not IBD, and the number of ROHs should be interpreted as a relative rather than an absolute measure. We therefore adapted the ROH criteria to the marker density following published guidelines [29]. Even in an analysis of Hanwoo ROHs using a 777k SNP chip [20], the average proportion of ROH was highest in the shortest class (0.5–1 Mb), which suggests that short ROHs are abundant in this breed irrespective of chip density; however, a direct test of the effect of density on our estimates was not performed.

The power of QTL mapping and GWAS, as well as the accuracy of genomic selection, depend on the extent of LD between SNP markers and QTLs [26,56]. According to [57], an LD of *r*^2^ ≥ 0.2 is required for genomic selection to achieve an accuracy of 85% in GEBV. Except for 10 chromosomes, the *r*^2^ value exceeded 0.2 when the distance between SNPs was 0–50 kb (Figure 6B). The average *r*^2^ value across autosomes was 0.228 at 0–50 kb, yet it decreased to 0.120 at 50–100 kb (Figure 5). Because the mean marker interval (59.7 kb) is larger than 50 kb, many adjacent SNP pairs fall in distance classes with *r*^2^ well below 0.2, so the marker density may limit the LD between markers and QTLs. An LD level of *r*^2^ ≥ 0.3 was suggested to increase power for QTL mapping and GWAS [6]; for chromosome 14, the average *r*^2^ was highest (0.312) at 0–50 kb (Figure 6B). Many QTLs that affect growth and carcass traits have been reported on chromosomes 6 and 14 in Hanwoo cattle [16,53,58]; both chromosomes also showed the two highest *D’* and *r*^2^ (Table 4), but whether this reflects selection was not tested.

In some studies of other cattle breeds, *r*^2^ was used because *D’* could be overestimated due to limited sample sizes or low allele frequencies [44,59,60]. Since few studies have used both *D’* and *r*^2^ for Hanwoo cattle, this study evaluated LD using both parameters. Studies on Hanwoo cattle consistently showed that LD was highest when SNPs were closest in physical distance and decreased rapidly as the distance between SNPs increased [40,41,42]. This pattern aligns with findings from other breeds including Eringer cattle [61], Limousin, Simmental, and Polish Red cattle [62], and Bali cattle [63]. Directly comparing and interpreting LD results between studies is challenging because of numerous factors, including sample size and marker types [64]. Nevertheless, the present study provides a basis for further research on genetic diversity in Hanwoo cattle.

Haplotype block structure can vary depending on genetic background and marker density. In this study, the number and length of haplotype blocks varied among chromosomes, with differences observed in the number of SNPs within the identified blocks. Most blocks (60.6%) contained only two SNPs, and the blocks covered only 3.53% of the autosomal genome, which reflects the marker spacing (mean 59.7 kb) and the 500 kb maximum distance; the blocks therefore describe the structure detectable with this chip rather than the complete haplotype structure of the genome. The largest numbers of haplotype blocks were found on chromosomes 6, 14, and 7 (245, 190, and 124). Chromosomes 6 and 14 have shown significant QTL associations with carcass traits such as carcass weight and backfat thickness in Hanwoo cattle [65,66,67]. In other breeds, specifically composite beef cattle breeds composed of Red Angus, Charolais, and Tarentaise cattle, SNPs associated with fat thickness have been reported on BTA14 [68]. In addition, QTLs associated with female fertility traits in cows have been reported in Nordic Red cattle [69]. Understanding the haplotype block structure can help in designing conservation programs, maintaining genetic diversity, and avoiding inbreeding [70].

The LD-based Ne method relies on correlations between alleles over distance, and low LD between distant SNPs can lead to underestimation of Ne [71]. In this study, Ne was estimated for the period from 1691 to 13 generations ago. Ne decreased from 2853 to 277, a decrease of about 10-fold, and the decline became steeper from about 180 generations ago. For interpretation, the history of the breed is relevant: Hanwoo has been used as a draft animal for at least 5000 years [42]. Its role changed to meat production from the 1960s [2], and progeny testing for bull selection began in the 1980s [72,73]. The generation interval of Hanwoo has been reported as 5.1–5.5 years in breeding herds [72] and as 4.3 years (dam-daughter) and 7.5 years (sire-daughter), with an average of 5.9 years, in a cow improvement population [73]. With an assumed generation interval of 5–6 years, 13, 100, 1000, and 1691 generations correspond to approximately 65–80, 500–600, 5000–6000, and 8500–10,000 years, respectively. Estimates older than about 1000 generations therefore refer to a period before the earliest records of Hanwoo as a draft animal and to the ancestral populations of the breed, and they should be interpreted with caution. Organized breeding since the 1960s covers only about the last 10 generations, so the decline that became steeper from about 180 generations ago (approximately 900–1100 years) largely predates modern breeding programs, and the present data cannot identify its cause. All estimates also depend on SNP density, sample structure, and the assumed recombination rate.

The Food and Agriculture Organization of the United Nations (FAO) states that a Ne of 50 is required for a stable population [74]. Moreover, ref. [75] suggested that the threshold Ne to maintain genetic diversity within a population is 100. The recent Ne value (277) is above these thresholds; however, because Ne has been decreasing, continuous monitoring and appropriate breeding programs are required to maintain genetic diversity. These Ne values describe the sampled cows and should not be taken as the Ne of the whole Hanwoo breed.

## 5. Conclusions

This study used the Hanwoo SNP50k BeadChip to analyze the genetic diversity and genomic structure of Hanwoo cows. Genetic diversity was assessed using various genetic parameters. We analyzed the ROHs in Hanwoo cows and confirmed their genomic structure by measuring LD and haplotype block structure. In addition, we found that the effective population size decreased from past to recent generations; heterozygosity did not decline across recent birth cohorts, but ROH-based inbreeding increased modestly. These findings are expected to provide valuable insights for future studies (GWAS and GS). Based on these results, the genetic diversity of Hanwoo cows should be continuously monitored, and appropriate breeding programs are required to preserve it.

## Figures and Tables

**Table 1:** Quality control of Hanwoo cows and SNPs.

Category	Quality Control Step	Removed (*n*)	Remaining (*n*)
Cows	Sample	-	7273
	Call rate < 0.90	68	7205
	Duplicate samples	39	7166
SNPs	All SNPs	-	53,866
	Autosomal SNPs	1671	52,195
	Call rate < 0.90	822	51,373
	Identical position	71	51,302
	MAF < 0.05	9418	41,884

SNP = single-nucleotide polymorphism; MAF = minor allele frequency.

**Table 2:** Summary of observed heterozygosity (H_O_), expected heterozygosity (H_E_), and minor allele frequency (MAF) by chromosome.

Chr.	SNP (*n*)	Observed Heterozygosity (H_O_)	Expected Heterozygosity (H_E_)	MAF
		Mean ± SD	Mean ± SD	Mean ± SD
1	2626	0.359 ± 0.124	0.358 ± 0.123	0.268 ± 0.131
2	2188	0.357 ± 0.126	0.357 ± 0.126	0.268 ± 0.133
3	2065	0.358 ± 0.125	0.358 ± 0.123	0.267 ± 0.130
4	1943	0.361 ± 0.126	0.360 ± 0.125	0.271 ± 0.133
5	1705	0.356 ± 0.127	0.355 ± 0.126	0.266 ± 0.133
6	2596	0.356 ± 0.127	0.355 ± 0.127	0.267 ± 0.135
7	2035	0.365 ± 0.123	0.364 ± 0.123	0.275 ± 0.131
8	1787	0.357 ± 0.124	0.357 ± 0.123	0.267 ± 0.131
9	1624	0.351 ± 0.127	0.350 ± 0.127	0.262 ± 0.135
10	1893	0.353 ± 0.129	0.352 ± 0.128	0.264 ± 0.135
11	1725	0.359 ± 0.126	0.358 ± 0.125	0.269 ± 0.133
12	1270	0.357 ± 0.125	0.356 ± 0.123	0.266 ± 0.131
13	1339	0.361 ± 0.123	0.361 ± 0.122	0.271 ± 0.129
14	1832	0.347 ± 0.120	0.346 ± 0.119	0.252 ± 0.126
15	1333	0.359 ± 0.123	0.359 ± 0.122	0.268 ± 0.130
16	1252	0.360 ± 0.124	0.359 ± 0.123	0.269 ± 0.131
17	1234	0.353 ± 0.126	0.352 ± 0.126	0.262 ± 0.132
18	1046	0.360 ± 0.124	0.359 ± 0.123	0.267 ± 0.129
19	1134	0.362 ± 0.122	0.362 ± 0.121	0.271 ± 0.129
20	1249	0.353 ± 0.128	0.353 ± 0.127	0.263 ± 0.133
21	1156	0.367 ± 0.123	0.366 ± 0.123	0.277 ± 0.132
22	977	0.353 ± 0.129	0.352 ± 0.128	0.263 ± 0.135
23	939	0.365 ± 0.122	0.365 ± 0.121	0.276 ± 0.131
24	1002	0.359 ± 0.124	0.358 ± 0.123	0.269 ± 0.132
25	792	0.367 ± 0.127	0.366 ± 0.126	0.280 ± 0.136
26	835	0.361 ± 0.122	0.361 ± 0.122	0.271 ± 0.131
27	758	0.363 ± 0.128	0.362 ± 0.127	0.274 ± 0.134
28	730	0.356 ± 0.127	0.355 ± 0.126	0.266 ± 0.133
29	819	0.360 ± 0.124	0.360 ± 0.123	0.271 ± 0.131
Mean	1444	0.358 ± 0.125	0.357 ± 0.124	0.268 ± 0.132

Chr = chromosome; SNP = single-nucleotide polymorphism; SD = standard deviation.

**Table 3:** Heterozygosity and ROH-based inbreeding by birth cohort.

Birth Cohort	Cows (*n*)	H_O_	H_E_	F_ROH (≥1 Mb)	F_ROH (≥4 Mb)
≤2011	226	0.352	0.353	0.0196	0.0114
2012	209	0.354	0.355	0.0208	0.0124
2013	259	0.356	0.355	0.0206	0.0122
2014	325	0.358	0.356	0.0196	0.0115
2015	484	0.357	0.356	0.0221	0.0141
2016	819	0.359	0.357	0.0223	0.0143
2017	1112	0.358	0.357	0.0218	0.0138
2018	1238	0.359	0.357	0.0243	0.0164
2019	1127	0.359	0.357	0.0256	0.0175
2020	1091	0.359	0.357	0.0264	0.0181
2021	198	0.357	0.356	0.0287	0.0202

ROH = runs of homozygosity; H_O_ = observed heterozygosity; H_E_ = expected heterozygosity; F_ROH = ROH-based inbreeding coefficient.

**Table 4:** Mean and standard deviation of *D’* and *r*^2^ by the number of SNP pairs for each chromosome.

Chr.	SNP Pairs (*n*)	*D’*		*r* ^2^	
		Mean	SD	Mean	SD
1	45,696	0.3710	0.2995	0.0487	0.1113
2	37,791	0.3564	0.2967	0.0460	0.1117
3	38,761	0.3817	0.3077	0.0453	0.1004
4	32,187	0.3778	0.3085	0.0506	0.1139
5	25,890	0.3867	0.3151	0.0468	0.1154
6	68,475	0.4315	0.3390	0.0778	0.1719
7	44,061	0.3270	0.2803	0.0440	0.1049
8	28,030	0.3629	0.2961	0.0433	0.0981
9	26,770	0.3914	0.3088	0.0517	0.1165
10	49,279	0.4012	0.3189	0.0552	0.1278
11	28,412	0.3529	0.2929	0.0403	0.0930
12	19,449	0.3405	0.2881	0.0364	0.0869
13	21,547	0.3624	0.2903	0.0435	0.0911
14	45,279	0.5759	0.3593	0.1510	0.2414
15	22,454	0.3637	0.2927	0.0429	0.0913
16	20,392	0.3533	0.2892	0.0436	0.1019
17	20,972	0.3608	0.2949	0.0426	0.0968
18	17,376	0.3375	0.2818	0.0376	0.0858
19	22,245	0.3370	0.2865	0.0400	0.0921
20	25,519	0.3655	0.2983	0.0489	0.1256
21	20,422	0.3705	0.2964	0.0489	0.1057
22	15,515	0.3574	0.2884	0.0374	0.0842
23	18,043	0.3472	0.2845	0.0446	0.1044
24	16,403	0.3350	0.2798	0.0379	0.0890
25	14,825	0.3560	0.2985	0.0389	0.0859
26	13,513	0.3431	0.2829	0.0399	0.0869
27	12,742	0.3439	0.2882	0.0388	0.0948
28	11,990	0.3583	0.2836	0.0370	0.0750
29	13,527	0.3407	0.2740	0.0365	0.0782
Total (mean)	777,565	(0.3807)	(0.3093)	(0.0538)	(0.1260)

Chr = chromosome; SNP = single-nucleotide polymorphism; SD = standard deviation.

**Table 5:** Summary statistics for the haplotype block structure of each chromosome.

Chr.	Blocks (*n*)	Total Block Length (kb)	Block Size (kb)			SNPs in Blocks (*n*)	SNPs (n)		
			Average ± SD	Max	Min		Average ± SD	Max	Min
1	95	5865.98	61.75 ± 71.19	360.93	0.080	324	3.41 ± 2.13	14	2
2	89	4514.85	50.73 ± 71.86	396.29	0.189	280	3.15 ± 1.93	14	2
3	84	4493.74	53.50 ± 79.97	451.21	0.012	265	3.15 ± 2.14	18	2
4	85	5278.08	62.10 ± 87.46	381.48	0.027	251	2.95 ± 1.38	8	2
5	50	3235.26	64.71 ± 97.77	461.16	0.083	183	3.66 ± 3.55	24	2
6	245	13,176.41	53.78 ± 80.61	493.76	0.192	844	3.44 ± 2.35	18	2
7	124	3494.77	28.18 ± 45.07	328.95	0.536	329	2.65 ± 1.04	7	2
8	39	2768.87	71.00 ± 76.01	284.56	0.085	134	3.44 ± 1.80	10	2
9	67	2467.51	36.83 ± 51.17	204.31	0.993	201	3.00 ± 1.87	12	2
10	90	2654.30	29.49 ± 53.26	373.62	0.114	328	3.64 ± 3.44	20	2
11	59	3732.06	63.26 ± 80.22	327.15	0.923	178	3.02 ± 1.28	7	2
12	39	2705.94	69.38 ± 87.75	366.10	0.238	116	2.97 ± 1.25	6	2
13	40	1636.68	40.92 ± 71.87	270.18	0.066	101	2.52 ± 1.06	6	2
14	190	12,401.21	65.27 ± 91.93	480.32	0.170	701	3.69 ± 2.68	15	2
15	45	2897.07	64.38 ± 110.47	487.16	0.124	139	3.09 ± 1.46	7	2
16	39	1414.60	36.27 ± 57.92	235.68	0.887	118	3.03 ± 1.80	10	2
17	42	1817.28	43.27 ± 89.42	498.03	0.165	117	2.79 ± 1.68	8	2
18	38	1492.15	39.27 ± 92.25	477.63	0.514	101	2.66 ± 1.83	12	2
19	52	2170.42	41.74 ± 63.47	251.57	1.015	156	3.00 ± 1.57	10	2
20	47	2060.06	43.83 ± 58.31	260.29	0.625	181	3.85 ± 3.06	16	2
21	43	1173.80	27.30 ± 41.23	173.91	0.904	127	2.95 ± 1.46	10	2
22	25	825.95	33.04 ± 57.87	234.65	0.247	63	2.52 ± 1.08	5	2
23	46	1411.57	30.69 ± 62.44	362.05	0.923	137	2.98 ± 1.86	11	2
24	35	1097.03	31.34 ± 56.74	233.38	0.064	84	2.40 ± 0.95	6	2
25	33	1029.71	31.20 ± 59.90	276.19	0.070	82	2.48 ± 0.87	5	2
26	21	1293.57	61.60 ± 79.81	222.76	0.282	63	3.00 ± 1.45	6	2
27	22	725.56	32.98 ± 46.12	151.97	0.152	67	3.05 ± 1.99	11	2
28	15	154.12	10.27 ± 9.65	29.98	0.627	32	2.13 ± 0.35	3	2
29	19	280.95	14.79 ± 19.89	92.80	0.032	40	2.11 ± 0.46	4	2
Total	1818	88,269.5	48.55 ± 75.32	498.03	0.012	5742	3.16 ± 2.11	24	2

Chr = chromosome; SD = standard deviation; SNP = single-nucleotide polymorphism. Total: sums for the number of blocks, total block length, and SNPs in blocks; mean ± SD were calculated over all 1818 blocks (not as the average of the chromosome means); max and min are over all blocks.

## Data Availability

The data presented in this study are available on request from the corresponding author.

## Abbreviations

The following abbreviations are used in this manuscript:

AI	Artificial insemination
BTA	*Bos taurus* chromosome
Chr	Chromosome
F	Inbreeding coefficient
F_ROH	ROH-based inbreeding coefficient
FAO	Food and agriculture organization of the united nations
G	Genomic relationship matrix
GEBV	Genomic estimated breeding value
GS	Genomic selection
GWAS	Genome-wide association study
H_E_	Expected heterozygosity
H_O_	Observed heterozygosity
IBD	Identical-by-descent
KPN	Korean Proven Bull number
LD	Linkage disequilibrium
MAF	Minor allele frequency
Ne	Effective population size
QC	Quality control
QTL	Quantitative trait loci
ROH	Runs of homozygosity
SD	Standard deviation
SNP	Single-nucleotide polymorphism
